# Supplementary material for: Factors influencing the use of multiple HIV prevention services among transport workers in a city in southwestern Uganda
Source: PLOS Glob Public Health. 2023 Mar 2;3(3):e0001350. doi: 10.1371/journal.pgph.0001350 (PMC10021771; doi:10.1371/journal.pgph.0001350)
Supplement: Attachment — Submitted filename: Response to Reviewers comments.docx [file pgph.0001350.s002.docx]

| **#** | **Comment** | **Response** | **line #** |
| --- | --- | --- | --- |
|  | **Reviewer 1** |  |  |
|  | Reviewer #1: This is a very interesting and well written paper. i have a few comments: | We thank the reviewer for this positive feedback, and for the insightful comments. |  |
| #1 | "Like in other sub-Saharan countries, the decline in the prevalence of 67 HIV is partly attributed to the use of HIV prevention services such as condom use, and 68 antiretroviral based prevention services."  => Yes and many deaths which results in a smaller numerator… you should mention this | Thank you very much for your observation. We acknowledge that there are many other reasons for the decline in the rates of HIV other than use the use of multiple HIV prevention services and this is reason catered for by the use of the word “partly”. We have rephrased it as *“Notwithstanding the high mortality rates among patients with acquired immune deficiency syndrome (AIDs), the decline in the prevalence of HIV is “partly” attributed to the use of HIV prevention services such as condom use, and antiretroviral based prevention services, like in many other sub-Saharan countries”* | LN 66-69 |
| #2 | in this cross sectional analysis odds ratios are used perhaps prevalence ratios would be more appropriate (the significant results would not change) using a modified poisson. ORs overestimated the RR in such studies | Thank you very much for your suggestion. We agree with the reviewer’s suggestion and we have re-analyzed the data using the modified Poisson regression and reported the prevalence ratios and their 95% confidence ratios. This was revised throughout the whole paper (abstract, analysis, results, and discussion) | LN 42, 282, 370-436 |
| #3 | "...the findings show that most of the participants 340 had tested for HIV and then used condoms (16.2%)," =>most and 16.2% seem to contradict each other 1 in 6! | Thank you very much for your observation. We have changed the word “most” to the “ a relatively low” | LN 342 |
| #4 | discussion “Nevertheless, our analysis indicates that across single use, most study participants used condoms 474 (32.2%) followed by voluntary counseling and testing (27.1%), and safe male circumcision (SMC, 475 17.3%). » =>again most and 27.1% i am struck about how low it is. please rephrase it hides the fact that it is very low! perhaps the discussion focusses on those who used different methods but perhaps emphasize the low use of any single method which is an important message too. | Thank you very much for your observation. We have changed the word “most” to the “relatively low” | LN 469 |
| #5 | perhaps trying to identify subgroups doing a PCA or MCA analysis would lead to insights how many dimensions drive variability (screeplot and the kaiser rule) what variables load on these dimensions and what does this capture... it may add to your study | Thank you very much for your advice. Since we did not measure our outcome of interest using a scale, we felt that running a PCA would not be appropriate. Above all the scales for knowledge, perceived HIV risk and stigma had been previously validated by their original authors, as supported by references #30, #31 and #32 in the methods section. |  |
|  | **Reviewer 2** |  |  |
|  | Reviewer #2: The manuscript is well written. There is the logical flow of ideas, and it is reader friendly. Keeps the reader glued to the end. You have provided a detailed description of all the methodological process to help other authors replicate the study. The discussion is well done. You gave a cautious overall interpretation of results considering objectives, limitations, multiplicity of analyses, results from similar studies, and other relevant evidence You have also discussed the limitations of the study, taking into account sources of potential bias. The conclusion is well written and reflect the major findings of the study. Congratulations and thank you for the extra effort put into this paper.  i have just two comments for you to address based on the recommendations of STROBE. | We thank the reviewer for the positive feedback and suggestions. |  |
| #1 | Please clearly emphasize the strength of this study. | Thank you very much for your observation. Under the strength and limitation sections. The following statement was added “ The study sheds light on the use of multiple HIV prevention services across the various transport workers tracking long, short, and medium distances unlike the previous studies. | 538-539 |
| #2 | Please discuss the generalizability of the study results. | Thank you very much for your observation. Under the strength and limitation sections. The following statement was also added. “The findings of the study can be generalized to countries in sub-Saharan Africa with similar health care and transport systems (with the three classes of drivers) as Uganda.” | 539-541 |
